# Probability cueing of distractor locations: both intertrial facilitation and statistical learning mediate interference reduction

**DOI:** 10.3389/fpsyg.2014.01195

**Published:** 2014-11-06

**Authors:** Harriet Goschy, Sarolta Bakos, Hermann J. Müller, Michael Zehetleitner

**Affiliations:** ^1^Department of Psychology, Ludwig-Maximilians-Universität MünchenMunich, Germany; ^2^Graduate School of Systemic NeurosciencesPlanegg-Martinsried, Germany; ^3^Department of Psychological Sciences, Birkbeck College, University of LondonLondon, UK

**Keywords:** attentional capture, probability cueing, visual search, statistical learning, intertrial facilitation

## Abstract

Targets in a visual search task are detected faster if they appear in a probable target region as compared to a less probable target region, an effect which has been termed “probability cueing.” The present study investigated whether probability cueing cannot only speed up target detection, but also minimize distraction by distractors in probable distractor regions as compared to distractors in less probable distractor regions. To this end, three visual search experiments with a salient, but task-irrelevant, distractor (“additional singleton”) were conducted. Experiment 1 demonstrated that observers can utilize uneven spatial distractor distributions to selectively reduce interference by distractors in frequent distractor regions as compared to distractors in rare distractor regions. Experiments 2 and 3 showed that intertrial facilitation, i.e., distractor position repetitions, and statistical learning (independent of distractor position repetitions) both contribute to the probability cueing effect for distractor locations. Taken together, the present results demonstrate that probability cueing of distractor locations has the potential to serve as a strong attentional cue for the shielding of likely distractor locations.

## INTRODUCTION

In our daily visual environment, objects tend to be unevenly distributed, that is, they are more likely to appear in certain regions and less likely to appear in other regions. Previous research has demonstrated that observers can take advantage of uneven distributions of object positions, so as to more quickly detect or discriminate objects at probable, as compared to less probable, locations (e.g., Shaw and Shaw, 1977; Geng and Behrmann, 2002, 2005; Fecteau et al., 2009; Druker and Anderson, 2010). This capability has lead to two strands of research questions: (i) is the change in performance for frequent locations due to statistical learning or to intertrial priming, and (ii), more recently, can observers learn to avoid locations which probably contain a distractor? In contrast to most previous studies, which used inefficient visual search, flanker, or single-item classification tasks, the present study transfers the probability cueing effect to efficient (pop-out) search, in which both the target as well as the distractor are very salient items. In particular, the research questions are: (i) is there evidence that in efficient visual search locations probably containing a distractor can be ignored; (ii) what is the underlying mechanism: statistical learning or intertrial priming?

First, we briefly review the general finding of the probability cueing effect; second, we summarize the debate about what mechanism is responsible for the behavioral effect: statistical learning or intertrial priming. Third, evidence for the avoidance of distractor locations is reviewed before, fourth, the rational of the present study is presented.

In general, the finding that observers can exploit uneven distributions of target locations to enhance search performance has been referred to as “location probability effect” (Miller, 1988) or “probability cueing effect” (Geng and Behrmann, 2002). The earliest reports go back to Shaw and Shaw (1977), who asked their observers to recognize a target letter which appeared with varying probabilities (25 vs. 10 vs. 5%) at different locations of the display. Recognition accuracy for the target letter was better at locations with a higher probability of containing the target (for similar reaction time data, see Shaw, 1978; see also Müller and Findlay, 1987). Miller (1988) observed probability cueing effects for both absolute spatial locations (i.e., screen positions) and relative spatial locations (i.e., positions within a configuration of items). While Miller (1988) reported these two modulations to be of similar magnitude, Hoffmann and Kunde (1999) argued that probability cueing effects are more strongly driven by relative, as compared to absolute, spatial locations (see also Chun and Jiang, 1998). In a visual search task, Geng and Behrmann (2002) asked their participants to discriminate (the identity of) a target letter presented among several non-target letters. The target appeared with 80% probability in one half of the display and with 20% probability in the other half. Participants were not explicitly instructed about this uneven distribution, and the majority did not report any awareness of it at the end of the experiment. Nevertheless, response times (RTs) were reduced for targets appearing in the more probable, as compared to the less probable, target region.

Second, although probability cueing effects of this kind have since been reported repeatedly within a variety of paradigms (e.g., Geng and Behrmann, 2005; Fecteau et al., 2009; Druker and Anderson, 2010), the mechanisms underlying the probability cueing effect are still subject to debate. Traditionally, probability cueing effects have been interpreted in terms of statistical learning, that is, the formation of location-specific stimulus expectancies that reflect the statistical likelihood of a target appearing at a specific location (or region) across a longer sequence of trials (e.g., Hoffmann and Kunde, 1999; Geng and Behrmann, 2002, 2005; Druker and Anderson, 2010). However, examinations of statistical learning in probability cueing paradigms have typically been confounded by short-term intertrial effects: if a target is more likely to appear at a particular location, the probability of cross-trial target repetition(s) at that location is also increased, facilitating performance. In fact, a host of studies have shown that repeating the target position on consecutive trials yields improved performance compared to positional changes (e.g., Maljkovic and Nakayama, 1996; Kumada and Humphreys, 2002; Kristjánsson et al., 2007; Geyer et al., 2010). Walthew and Gilchrist (2006) have argued that target position repetitions of this kind, as opposed to statistical learning, are the underlying mechanism of the probability cueing effect. In their experiment, the target was more likely to appear on one side of the display compared to the other. In addition, there were two (between-subjects) repetition conditions: for the “repeat” group, target position repetitions were not restricted; for the “non-repeat” group, by contrast, there were no repetitions of the target position within a sequence of four trials. A probability cueing effect was observed only for the repeat group, but not for the non-repeat group; that is, when target position repetitions were restricted, there was no “statistical learning” effect. Also Kabata and Matsumoto (2012) failed to find a probability cueing effect, when repetitions of the target location were completely or partially absent.

However, the conclusion that the probability cueing effect is not a result of statistical learning but solely attributable to intertrial location priming is not unanimously accepted. For instance, Jones and Kaschak (2012) report a probability cueing effect in an inefficient search task, even in the absence of repetitions. Jiang et al. (2013b), also in an inefficient search task, segmented the experiment into two blocks. During the first block, one region in the display was more likely to contain the target. During this phase of the experiment, the more probable region naturally also contained more repetitions of the target location than the other regions. In the second – test – phase of the experiment, targets were equally likely in all display regions and, thus, target location repetitions too were equally likely in all regions. However, still, RTs were facilitated for the previously frequent region, indicating that statistical learning had indeed taken place. Druker and Anderson (2010), in a classification task, also reported a probability cueing effect independent of location priming. Note that statistical learning and intertrial facilitation as underlying mechanisms of the probability cueing effect are not necessarily mutually exclusive: for instance, recent work by Kabata and Matsumoto (2012) suggests that both statistical learning and intertrial facilitation contribute to the probability cueing effect, but that learning the target location probability is mediated by target location repetitions on consecutive trials.

Third, statistical regularities in the studies mentioned above always concerned the target. However, there are reasons to assume that search performance is influenced not only by statistical properties of the target, but also by statistical properties of possible distracting stimuli. For instance, interference by salient but irrelevant distractors (i.e., RT slowing in the presence compared to absence of a distractor) varies in magnitude as a function of distractor prevalence, with relatively little interference when distractors are frequent and substantial interference when distractors are rare (Forster and Lavie, 2008; Geyer et al., 2008; Müller et al., 2009; Zehetleitner et al., 2009; Sayim et al., 2010). Reder et al. (2003) investigated probability cueing of distractor positions in a target localization task. In their experiments, observers had to indicate which of four locations contained a target item (“o”), while a distractor item (“x”) could be present at the same time at one of the other locations. Critically, the distractor was not equally probable at those locations, which influenced RT performance: while distractors at frequent locations caused essentially no RT interference, distractors at rare locations produced considerable RT slowing. However, in a similar paradigm, there was no evidence of location-specific distractor suppression: Kelley and Yantis (2009) reported that, with practice, a highly salient onset distractor ceased to interfere with the required classification judgment. But, when the distractor was presented at a constant location for half of the experiment and interference had ceased, a change in distractor location did again result in behavioral interference. At first glance, this pattern suggests a probability cueing effect for the distractor position. However, at odds with this are the results from another condition in which the distractor was also presented at a constant location for half the experiment and then changed its identity (from a face to a colored disk, or vice versa), but not its location. If participants had learnt, in the first half of the experiment, to ignore the distractor location, there should be no interference after a change of the distractor identity at the same location. But, at variance with this expectation, an identity change did also induce a recurrence of distractor interference. Thus, there is no unanimous evidence for attentional shielding from locations likely to contain a distractor. Additionally, Reder et al. (2003) did not specifically examine the mechanism(s) underlying probability cueing. Hence, their design does not exclude the possibility of transitory (i.e., short-term) adjustments and cross-trial carry-over of control settings minimizing the effects of distractors appearing at repeated *positions* (analogous to the effects of cross-trial distractor *dimension* repetitions described by Müller et al., 2009) being the critical factor; in fact, as there were only four possible display locations (at which distractors occurred with unequal probabilities), distractor position repetitions would have been rather frequent.

In summary, probability cueing of distractor locations has hitherto only been investigated by two studies, with conflicting results. Given this, the present study was designed to investigate, first, whether a distractor probability cueing effect could be firmly established and, second, if found, what mechanism is responsible for its occurrence: statistical learning or probability cueing. To this end, we implemented a visual search task in which observers had to search for a target item surrounded by several non-targets. In a certain proportion of the trials, a task-irrelevant but salient distractor was presented (“additional-singleton paradigm”; Theeuwes, 1992), and distraction was operationalized as RT interference in the presence vs. the absence of this distractor. Experiment 1 was intended to, first of all, demonstrate probability cueing of distractor locations in a classical visual search paradigm, that is: would distractors at frequent locations cause less interference (i.e., RT slowing) than distractors at rare locations? Experiment 2 then investigated the contribution of cross-trial effects (i.e., distractor position repetitions) to interference reduction, that is: is it easier to ignore a distractor appearing at a location that had just recently contained a distractor? Finally, Experiment 3 was designed to examine whether distractor position repetitions are a prerequisite for the probability cueing effect for distractor locations, that is: would statistical learning also occur if distractor position repetitions are excluded by the experimental design?

## EXPERIMENT 1

Experiment 1 was designed to examine whether probability cueing of distractor locations can be used to selectively down-modulate interference by salient but irrelevant distractors (i.e., RT slowing on distractor-present, as compared to distractor-absent, trials) in a classical visual search task with orientation-defined targets and color-defined distractors. If this were the case, distractors at frequent distractor locations should cause less interference compared to distractors at rare distractor locations. Note that Experiment 1 was not yet meant to address the mechanism underlying this (possible) interference reduction (statistical learning, intertrial facilitation, or both), but rather to simply demonstrate the general effect in the present paradigm.

In contrast to Reder et al. (2003), who demonstrated a probability cueing effect on distractor interference in a target localization paradigm, we used frequent and rare distractor areas instead of single (absolute) distractor positions with different probabilities: if present (50% of the trials), the distractor appeared with a probability of 90% at one of the positions within the frequent distractor area, and with a probability of 10% at one of the positions within the rare distractor area. The target, which was present on every trial, appeared with equal probability in both distractor areas. The frequent vs. rare distractor area was either the left vs. the right hemifield, or, for a different group of observers, the bottom vs. the top hemifield. Distractor position repetitions were not restricted by the experimental design.

### METHOD

#### Participants

Twenty-five (19 female, 23 right-handed) observers with a median age of 22 years (range: 19–42 years) participated in this experiment. All of them reported normal or corrected-to-normal visual acuity and color vision. They were randomly assigned to the left/right group (*n* = 13) or the top/bottom group (*n* = 12).

#### Stimuli

The stimulus display, presented on a black background, consisted of gray (RGB: 127, 127, 127; CIE [Yxy]: 13.6, 0.28, 0.32) vertical bars (0.25° of visual angle wide, 1.35° high) whose geometric centers were equidistantly arranged on the circumferences of three concentric (imaginary) circles, with radii of 2, 4, and 6° and encompassing 6, 12, and 18 bars, repectively; a further gray bar occupied the position in the center of the three circles. In every bar, there was a gap 0.25° in height, which was randomly located 0.25° from the top or the bottom of the bar. The target differed from the non-targets by its unique orientation: in a random half of the trials, it was tilted 12° to the left, in the other half 12° to the right (orientation = target-defining property). Participants’ task (see below) was to respond to the position of the gap, top vs. bottom, in the target bar (gap position = response-defining property). If a distractor was present, one of the non-targets was red (RGB: 252, 0, 21; CIE [Yxy]: 14.2, 0.62, 0.34). The target and, if present, the distractor could appear only on the middle circle.

#### Apparatus

The experiment was conducted in a sound-isolated, dimly lit cabin with black interior. The search displays were presented on a monitor (22-inch Mitsubishi Diamond Pro®; 2070SB, refresh rate of 120 Hz, resolution of 1, 024 × 768 pixels), which observers viewed from a distance of about 70 cm. Stimuli were generated using a ViSaGe system (Cambridge Research Ltd., UK) and the Experimental Toolbox (Reutter and Zehetleitner, 2012) for MATLAB®; (The MathWorks®; Inc.), controlled by a personal computer running under the Windows XP®; operating system. The observers were asked to report whether the target bar had a gap at the top or the bottom by pressing the “Z” or the “M” key of a QWERTY keyboard (Empirisoft DirectIN, Empirirsoft Corporation, USA) using the index finger of their left and right hands, respectively.

#### Design

**FIGURE 1:**
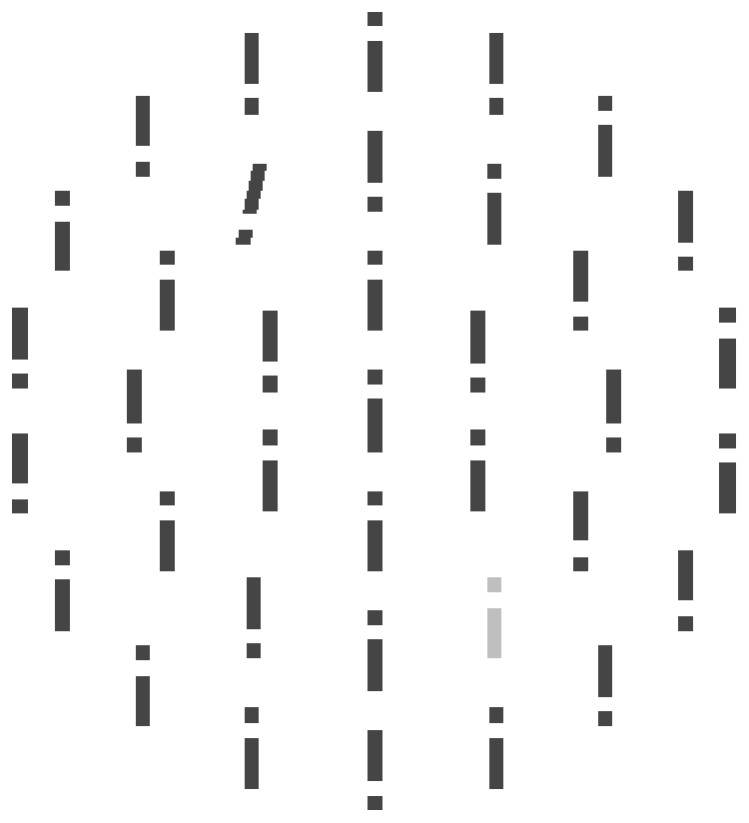
**Illustration of a stimulus display: the target item was defined by orientation and tilted 12° to the left or to the right.** The distractor was defined by color: if a distractor was present, one of the non-target items was red (light-gray in the example). The observers’ task was to indicate whether the target bar had a gap at the top or at the bottom.

The experiment consisted of 800 trials presented in eight blocks of 100 trials. Distractors were present in a random half of the trials (50 trials per block). The frequency distribution of the distractors was introduced as a between-subjects factor. For the left/right group, the frequent vs. rare distractor area was the left vs. right hemifield, that is, the range from the 7 o’clock to the 11 o’clock position vs. the 1 o’clock to the 5 o’clock position on the middle display circle (see **Figure 1**). For the top/bottom group, the frequent vs. rare area was the top vs. the bottom hemifield, that is, the range from the 10 o’clock to the 2 o’clock position vs. the 4 o’clock to the 8 o’clock position (see **Figure 1**). In the left/right group, neither the target and nor distractor ever appeared at the 12 and 6 o’clock positions, as these positions could not be assigned to either the left or right hemifield (i.e., the frequent or rare area), respectively. The same was the case for the top/bottom group and the 3 and 9 o’clock positions. The assignment of frequent and rare areas to the left and right hemifields (or to the top and bottom hemifields, respectively) was counterbalanced between participants.

If a distractor was present, it appeared, within each trial block, with 90% probability in the frequent hemifield and with 10% probability in the rare hemifield. That is, of the 50 distractor trials per block, there were 45 trials with a distractor in the frequent area (nine per frequent distractor position) and five with a distractor in the rare area (one per rare distractor position). Also, the target appeared equally often in both hemifields, with an equal probability for all ten possible positions. However, it never co-occurred with the distractor at one-and-the-same position, that is, there was never a red tilted bar. Trial presentation order within the blocks was randomized.

#### Procedure

Prior to the experiment, all observers received both written and oral instructions: their task was to indicate whether the target bar had a gap at the top or at the bottom and to proceed as fast and yet as accurately as possible. They were informed that on some trials, one of the non-targets would be red, which would be irrelevant to their task. However, they were not informed about the manipulation of distractor location probability.

Each trial started with the presentation of a white fixation cross (0.5° × 0.5°) in the center of the screen for a random duration between 700 and 1100 ms. Thereupon the search display appeared and remained visible until the observer’s key press response. If the response was correct, a new trial began; if the response was incorrect, the word “Fehler” (German for error) was presented in the center of the screen for 500 ms before a new trial started. After each block of trials, observers were informed about their average RT and their percentage error rate in the previous block via a message on the screen. Observers could take short breaks between blocks of trials and started each block by a button press.

Subsequently to performing the experiment, participants were debriefed and asked to indicate whether the additional singleton had occurred more frequently in one part of the display (response alternatives: upper, lower, left, or right half) or equally frequently in all parts (response option: equal).

### RESULTS

RTs more than three standard deviations above an observer’s mean per distractor presence condition (present vs. absent) and below 200 ms were discarded as outliers (1.83% of all trials). Subsequently, error trials were excluded as well (5.64% of all trials). Mean error rates did not differ significantly depending on whether the distractor was absent (5.55%), or appeared at a rare position (6.85%) or at a frequent position (5.48%), *F*(1.19,28.58) = 2.46, MSE = 10.24, *p* = 0.12, *ns* (Greenhouse-Geisser-corrected values). Accordingly, a speed-accuracy trade-off influence on the RTs can be ruled out.

**FIGURE 2:**
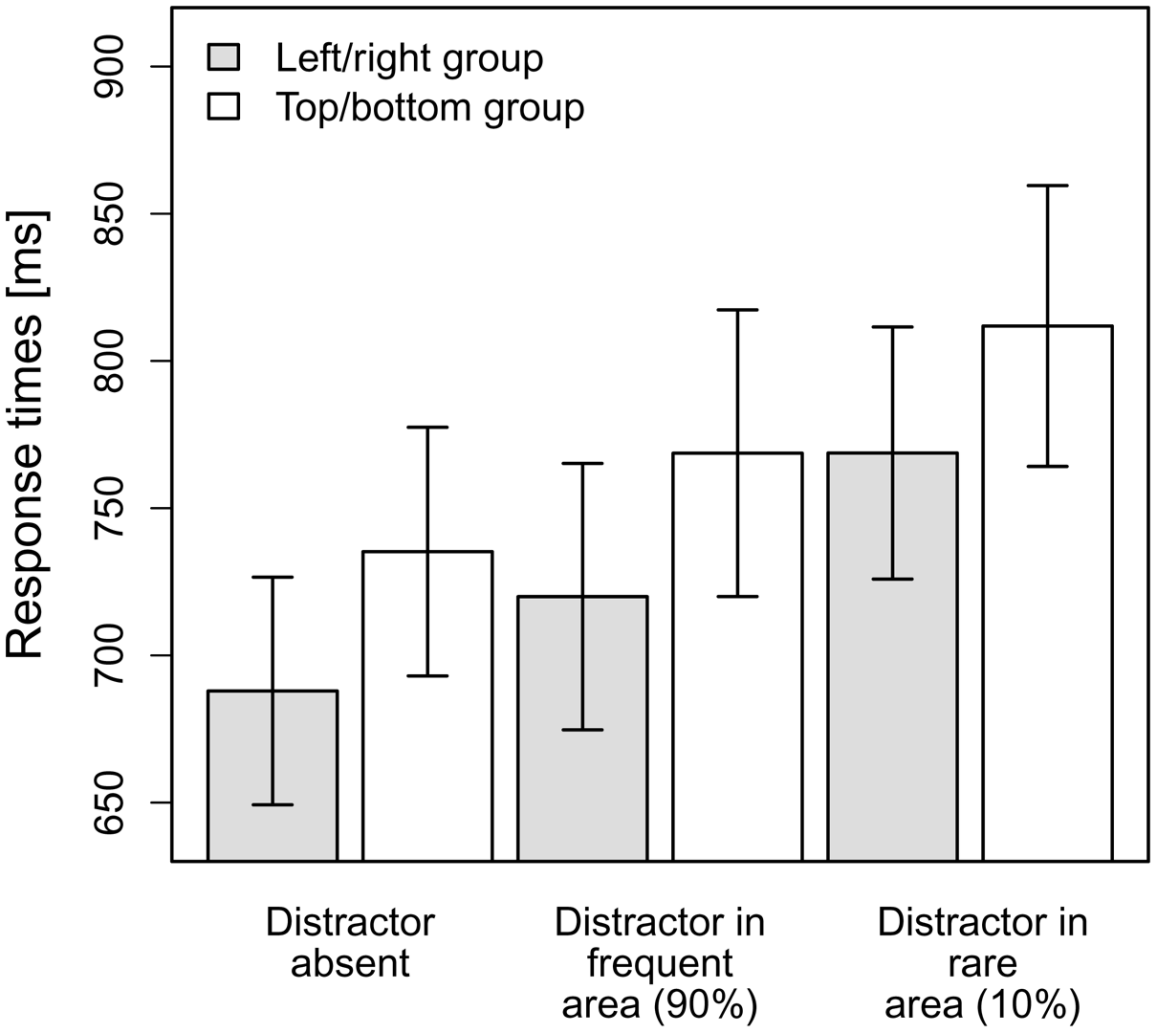
**Mean RTs for the top/bottom group and the left/right group dependent on the distractor condition in Experiment 1.** Error bars denote one standard error of the mean RT.

The mean RTs per observer and condition were entered into a 2x3-ANOVA with the between-subjects factor distractor frequency distribution (left/right vs. top/bottom) and the within-subjects factor distractor condition (distractor-absent, distractor in frequent area, distractor in rare area). As can be seen in **Figure 2**, the top/bottom group exhibited numerically slower overall RTs (*M* = 754 ms, SD = 155) than the left/right group (*M* = 706 ms, SD = 147); however, this difference was not significant [non-significant main effect of distractor frequency distribution, *F*(1,23) = 0.56, MSE = 71,646.69, *p* = 0.46, *ns*]. The main effect of distractor condition was significant, *F*(1.33,30.62) = 46.27, MSE = 1,267.24, *p* < 0.001, $\eta_{p}^{2}$ = 0.67 (Greenhouse-Geisser-corrected values), and is evident for both the top/bottom group and the left/right group, as indicated by a non-significant interaction effect between distractor frequency distribution and distractor condition, *F*(1.33,30.62) = 0.06, MSE = 1,267.24, *p* = 0.87, *ns* (Greenhouse-Geisser-corrected values; see also **Figure 2**).

As there was no significant interaction effect, we further analyzed the main effect distractor condition, irrespective of the distractor frequency distribution, via planned (orthogonal, one-tailed) *t*-tests according to our hypotheses. The first comparison tested whether there was a significant overall-interference effect caused by the presence of distractors, by comparing distractor-absent RTs to the averaged RTs for the conditions “distractor in frequent area” and “distractor in rare area.” This comparison turned out to be significant, that is, RTs were overall slower when a distractor was present, *t*(24) = 7.77, *p* < 0.001, *d* = 1.55. The second comparison contrasted the two distractor-present conditions, revealing that RTs were indeed significantly faster if a distractor appeared at a frequent position as compared to a rare position, *t*(24) = –5.90, *p* < 0.001, *d* = 1.18. As can be seen in **Figure 2**, the interference caused by a distractor in the frequent area (33 ms) was considerably smaller [though significantly different from zero, *t*(24) = 6.27, *p* < 0.001, *d* = 1.25] than that produced by a distractor in the rare area (79 ms)^1^.

In a final analysis, we examined whether the reduced interference by distractors at frequent vs. rare distractor locations is critically dependent on participants having “recognized” the distractor distribution. This analysis was based on participants’ responses to the (post-experimental) query whether the distractor had occurred more frequently in one part of the display (response alternatives: upper, lower, left, or right half) or equally frequently in all parts (response option: equal). For 8 of the 25 participants (32%), the response given tallied with the actual location of the distractor; 11 of the 25 participants (44%) responded “equal.” Comparing performance between participants who had (eight participants) vs. those who had not (17 participants) correctly indicated the frequent distractor region, in a group × distractor condition analysis of variance (ANOVA), revealed [besides a significant distractor condition main effect: *F*(1.37,31.52) = 38.12, *p* < 0.001; Greenhouse-Geisser-corrected values] a marginally significant interaction, *F*(1.37,31.52) = 2.38, *p* = 0.063 (Greenhouse-Geisser-corrected values). For those who had not correctly indicated the frequent distractor region, the singleton distractor produced an interference effect of 34 ms if it appeared at one of the frequent locations, and an effect of 91 ms if it appeared at one of the rare locations. This compares with effects of 29 ms and, respectively, 52 ms for participants who had correctly indicated the frequent distractor region. Thus, if anything, the (marginal) interaction was driven by differential interference for rare positions (91 vs. 52 ms) – making it unlikely that the reduced interference for frequent positions (34 vs. 29 ms) was influenced by whether or not participants “recognized” the distractor distribution.

### DISCUSSION

In Experiment 1, interference by a salient but irrelevant distractor was reduced if it appeared at a frequent, as compared to a rare, distractor location. Hence, the present results demonstrate that probability cueing cannot only directly speed up target detection, but can also serve to reduce interference by salient but irrelevant distractors (i.e., to facilitate distractor suppression) in visual search. In this regard, the present results are in line with Reder et al. (2003), who observed a similar interference modulation in a target localization paradigm. However, unlike Reder et al. (2003), we did not manipulate positional distractor probability between single (absolute) distractor positions, but between different distractor areas (encompassing several frequent or rare distractor positions). Given this, the present results imply that distractor shielding based on probability cueing of distractor positions, does not only reduce interference for single (precisely defined) distractor positions, but can also extend to larger display areas comprising several distractor positions.

Note that the presently observed effect is not primarily attributable to (cerebral) hemisphere-specific selectivity adaptation, with each hemisphere adopting an appropriate processing strategy independently of the other hemisphere’s strategy: we observed no significant interaction between the distractor condition and the distractor frequency distribution (left/right vs. top/bottom). If the observed interference modulation effect were primarily attributable to hemisphere-specific selectivity adjustment, it should have been evident only in the left/right group (in which frequent and rare distractors were presented in different visual hemifields), but not in the top/bottom group (in which both frequent and rare distractors were presented in both hemifields). Hence, the interference modulation observed in Experiment 1 is likely the result of a location-specific selectivity adjustment, and by and large independent of hemisphere-specific processing. In this regard, the present results are in line with a variety of findings in the cognitive control literature, where independent effects of the ratio of congruent and incongruent trials for different stimulus locations were reported that were also not based on hemisphere-specific selectivity (Crump et al., 2006; Wendt et al., 2008; but see also Corballis and Gratton, 2003, for a more hemisphere-specific selectivity account).

Concerning the question whether the probability cueing of distractor locations revealed in Experiment 1 is “implicit” vs. “explicit” in nature, the results were reasonably clear: there was no evidence that the reduced interference by distractors in frequent distractor locations is critically dependent on participants having “recognized” the actual distractor distribution. Given this, it is likely that the reduced interference reflects an “implicit” learning effect.

The results of Experiment 1 demonstrated that probability cueing of distractor locations enables a selective, location-specific down-modulation of interference by salient but irrelevant distractors. However, Experiment 1 does not permit any conclusions to be drawn about the mechanism(s) underlying this effect: (longer-term) statistical learning, (short-term) cross-trial adjustments, or both. To disentangle these effects, Experiment 2 investigated the contribution of intertrial adjustments independently of statistical learning, while Experiment 3 investigated the contribution of statistical learning independently of intertrial adjustments.

## EXPERIMENT 2

Experiment 2 was designed to investigate the contribution of cross-trial facilitation of distractor suppression (independently of statistical learning) to the probability cueing effect for distractor locations established in Experiment 1, that is: is it easier to ignore a distractor at a just encountered distractor location? In the light of previous studies, there is reason to assume that intertrial facilitation (i.e., repeating the distractor position from trial n-1 to trial n) might have contributed to the reduction of distractor interference observed in Experiment 1. For instance, examining distractor interference in a visual search task, Kumada and Humphreys (2002) found RTs to be slowed if a target on the current trial n appeared at a position occupied by a singleton distractor on the preceding trial n-1, which they interpreted in terms of “negative position priming.” If a position previously occupied by a singleton distractor is inhibited (and if the inhibitory tag persists for a while), this should also affect singleton distractors subsequently appearing at that position, resulting in reduced distractor interference. For repetitions of target locations, there is a large body of evidence demonstrating a facilitation of performance when the target location is repeated (e.g., Maljkovic and Nakayama, 1996; Müller et al., 2009). For distractors, by contrast, the role of location repetition has, to our knowledge, never been investigated.

Experiment 2 was similar to Experiment 1, except that there was no spatial probability manipulation. Instead, both the target and the distractor appeared equally often at one of six different positions of the search display, and RTs were analyzed as a function of the intertrial transitions from trial n-1 to trial n. If there is a contribution of intertrial facilitation, we expected distractor interference to be smaller for distractor position repetitions from trial n-1 to trial n as compared to distractor position switches. In addition, based on previous findings (Müller et al., 2009; Zehetleitner et al., 2009), we expected interference on distractor-present trials (trial n) to be larger following distractor-absent trials (trial n-1) compared to both distractor position repetitions and switches, owing to increased recruitment of attentional control following the (recent) encounter of distraction on the preceding trial (see also Botvinick et al., 2001).

### METHOD

Experiment 2 was methodologically identical to Experiment 1, with the following exceptions.

#### Participants

Twelve (10 female, all right-handed) new observers with a median age of 25.5 years (range: 20–40 years) participated in Experiment 2.

#### Design

The experiment consisted of 720 trials presented in 12 blocks of 60 trials. Distractors were present in a random half of the trials (30 trials per block). To ensure a sufficiently large number of distractor position repetitions, there were only six possible distractor positions: the distractor, if present, appeared equally often at the 1, 3, 5, 7, 9, and 11 o’clock positions of the middle display circle (each five times per block). Likewise, the target appeared only, and equally frequently, at one of these positions (10 trials per block). On the one hand, possible distractor and target positions were restricted to those six positions to ensure a sufficient number of position repetition trials. On the other hand, there is also evidence that distractor inhibition might spread spatially to neighboring positions (Kumada and Humphreys, 2002). By never presenting the target and (if present) the distractor at directly adjacent positions, we tried to avoid possible confounding influences of such spreading positional inhibition. Also, as previously, the target and distractor could not co-occur at one and the same position. Trial presentation order within the blocks was randomized^2^.

### RESULTS

RTs more than three standard deviations above the individual observer’s mean per distractor presence condition (present vs. absent) and below 200 ms were discarded as outliers (overall, 1.67% of trials). Subsequently, error trials were excluded from the analysis (4.68% of all trials). For data analysis, the trials were sorted into four categories dependent on distractor presence and distractor position on the previous trial n-1 and distractor presence and distractor position on the current trial n: (1) distractor-absent on trial n (irrespective of distractor presence on trial n-1); (2) distractor-present on both trial n and n-1 with a distractor position repetition; (3) distractor-present on both trial n and n-1 with a distractor position switch; (4) distractor-present on trial n, but absent on trial n-1. The first trial of each block was excluded from the analysis, as it was impossible to assign it to a category. After data filtering, the critical distractor position repetition category – with the fewest trials – included on average 22.5 trials per participant (minimum 16 trials). Mean error rates did not differ significantly depending on the distractor condition, *F*(3,33) = 0.53, MSE = 3.62, *p* = 0.67, *ns*. Thus, a speed-accuracy trade-off can be ruled out for the RT data.

**FIGURE 3:**
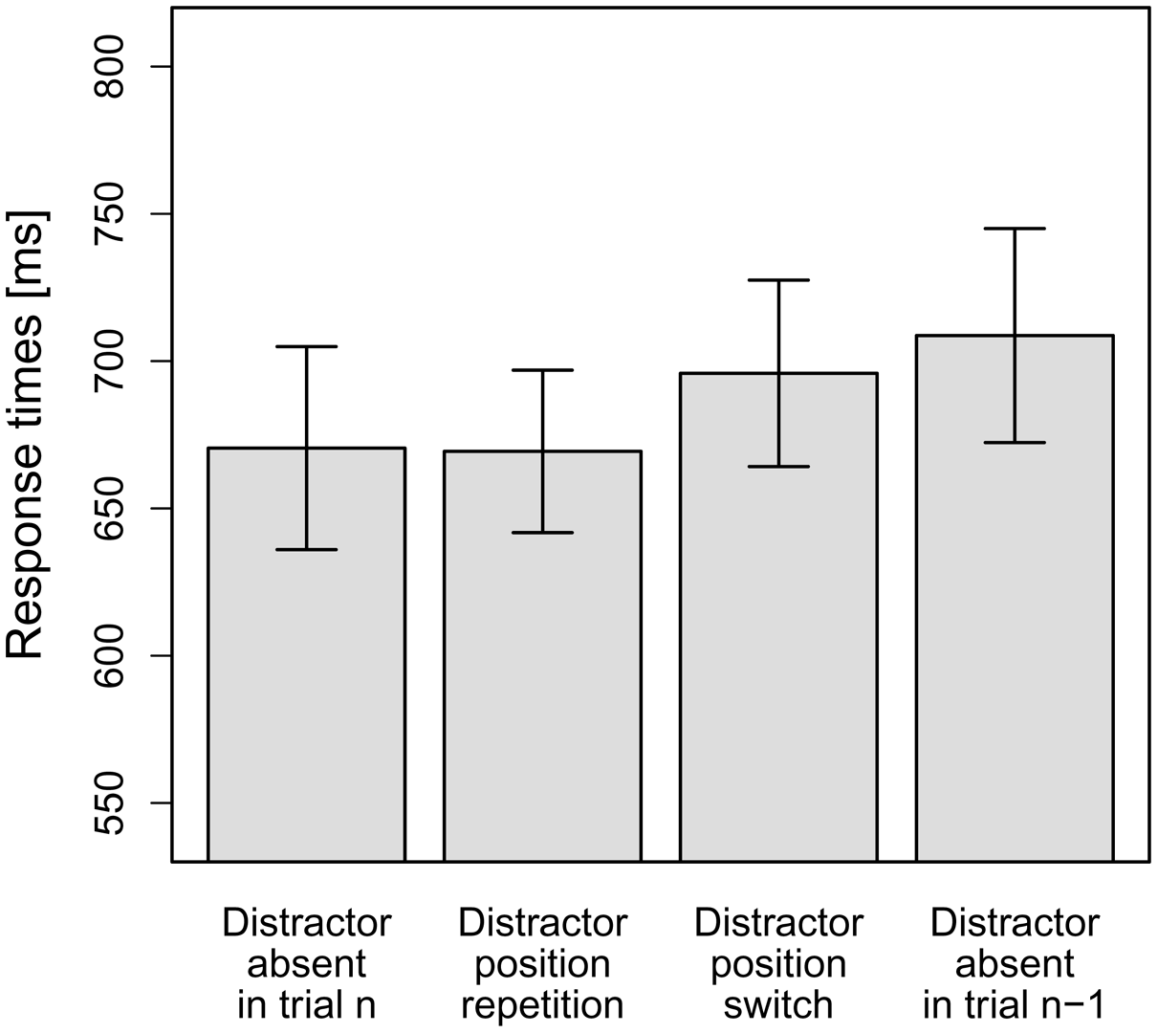
**Mean RTs for the four distractor conditions in Experiment 2: distractor-absent in the current trial n, distractor position repetition from the previous trial n-1 to the current trial n, distractor position switch from n-1 to n or distractor-absent in trial n-1, but present in n.** Error bars denote one standard error of the mean RT.

As can be seen in **Figure 3**, in line with our hypotheses, there was effectively zero interference on distractor position repetition trials (–1 ms). By contrast, interference was increased on distractor position switch trials (25 ms), and was even larger for distractor-present trials following distractor-absent trials (38 ms). To statistically examine this pattern, the mean RTs per participant and distractor condition were entered into a repeated-measures ANOVA, which revealed the main effect to be significant, *F*(1.51,16.57) = 5.84, MSE = 1,539.52, *p* = 0.018, $\eta_{p}^{2}$ = 0.35 (Greenhouse-Geisser-corrected values). Again, to test our hypotheses, we conducted planned (orthogonal, one-tailed) *t*-tests to break down this effect. The first comparison tested whether the overall-interference effect was significant. To this end, distractor-absent RTs were compared to the averaged RTs for the three distractor-present conditions. Indeed, distractor-present RTs were significantly slower overall than distractor-absent RTs, *t*(11) = 2.54, *p* = 0.014, *d* = 0.73. The second comparison tested whether RTs for distractor-present trials were significantly slower if trial n-1 was a distractor-absent trial, as compared to a distractor-present trial. To this end, the RTs for the condition “distractor-present on trial n but absent on trial n-1” were compared to the averaged RTs for the other two distractor-present conditions (“distractor position repetition” and “distractor position switch” conditions). This comparison revealed a significant difference, *t*(11) = 2.52, *p* = 0.014, *d* = 0.73. Finally, we tested whether RTs for distractor position repetition trials were significantly faster than RTs for distractor position switch trials, which was the case, *t*(11) = 2.22, *p* = 0.024, *d* = 0.64.

### DISCUSSION

Experiment 2 yielded a significant distractor interference effect (comparison 1), with interference on trial n being significantly reduced if a distractor was present vs. absent on trial n-1 (comparison 2). This replicates previous findings (Müller et al., 2009; Zehetleitner et al., 2009) and is in line with the assumption that a conflict encounter (i.e., a distractor-present trial) leads to increased recruitment of cognitive control, which helps to resolve subsequent conflict encounters (Botvinick et al., 2001).

Most importantly, RTs on distractor-present trials following distractor-present trials were significantly faster if the distractor position was repeated rather than switched (comparison 3). Hence, we observed significant intertrial facilitation by distractor position repetitions: observers could more effectively control for distractor interference following distractor position repetitions compared to distractor position switches. In fact, responding on distractor position repetition trials was not slowed at all compared to distractor-absent trials (–1 ms), that is, interference was completely eliminated following a distractor position repetition. It should be emphasized that this was the case despite a distractor re-appearing at the same position was just as likely as it appearing at any other distractor position, that is: there was no specific incentive to shield from interference arising at a recent distractor position.

Taken together, the results of Experiment 2 demonstrate that distractor position repetitions are associated with cross-trial facilitation of distractor suppression: interference from singleton distractors can be down-regulated more effectively if the position of a distractor repeats from trial n-1 to trial n, as compared to a position switch. As we did not prevent distractor position repetitions in Experiment 1 and the rate of repetitions was higher for the frequent, as compared to the rare, distractor area, the probability cueing effect observed in Experiment 1 is at least partly attributable to intertrial facilitation on distractor position repetitions^3^. Given this, the goal of Experiment 3 was to investigate whether statistical learning – in addition to intertrial facilitation – might have contributed to the probability cueing effect in Experiment 1, or whether this effect was attributable solely to intertrial facilitation.

## EXPERIMENT 3

The objective of Experiment 3 was to examine whether statistical learning can be observed also in the absence of facilitation by distractor position repetitions. Accordingly, Experiment 3 was basically a replication of Experiment 1 – however, the experimental design prevented distractor position repetitions from trial n-1 to trial n (and from trials preceding trial n-1 if all intervening trials were distractor-absent trials)^4^. If the probability cueing effect for distractor locations is solely attributable to intertrial facilitation, no such effect should manifest under the conditions of Experiment 3. However, if statistical learning contributes to the probability cueing effect, reduced distractor interference should also be observable even without distractor position repetitions (which were eliminated in Experiment 3).

### METHOD

Compared to Experiment 1, only the following methodological changes were made in Experiment 3.

#### Participants

Twenty (13 female, 19 right-handed) new observers with a median age of 25.5 years (range: 19–46 years) participated in Experiment 3.

#### Design and procedure

The experiment consisted of 720 trials presented in 12 blocks of 60 trials. Distractors were present in a random half of the trials (30 trials per block). Both targets and distractors appeared only on the 1, 3, 5, 7, 9, and 11 o’clock positions of the middle display circle (as in Experiment 2). The target appeared equally often on each of these positions. If a distractor was present, it appeared with 90% probability in the frequent hemifield (27 trials per block, with nine trials per possible position) and with 10% probability in the rare hemifield (three trials per block, with one trial per possible position). Again, target and distractor never co-occurred on one and the same position. For a random half of the participants, the right hemifield was the frequent hemifield and the left hemifield the rare hemifield, and vice versa for the other half of the participants. The design included the following restriction to exclude the influence of distractor position repetitions: the distractor position never repeated on two successive distractor-present trials – regardless of how many distractor-absent trials intervened between the two distractor-present trials (e.g., if trial n-4 was a distractor-present trial and trials n-3, n-2, and n-1 were distractor-absent trials, a distractor on trial n would not appear at the position of the distractor on trial n-4).

Further, in Experiment 3, participants were again queried about their explicit knowledge of the positional distribution of the distractor stimuli. In contrast to Experiment 1, this involved a two-stage procedure: first, participants were asked whether they had noticed anything about the “positional distribution of the red singleton distractor”; second, following their response to query 1, participants were asked to indicate in which display half the distractor had appeared more frequently (response alternatives: left, right, don’t know).

### RESULTS AND DISCUSSION

RTs more than three standard deviations above the individual observer’s mean per distractor presence condition (present vs. absent) and below 200 ms were excluded from the analysis (overall, 2.34% of trials), as were error trials subsequently (4.58% of all trials). Mean error rates did not differ significantly depending on whether a distractor was absent (4.33%) or appeared in the rare hemifield (4.94%) or the frequent hemifield (4.20%), *F*(1.14,21.64) = 0.59, MSE = 9.30, *p* = 0.47, *ns* (Greenhouse-Geisser-corrected values). Consequently, a speed-accuracy trade-off can be ruled out for the RT data.

**FIGURE 4:**
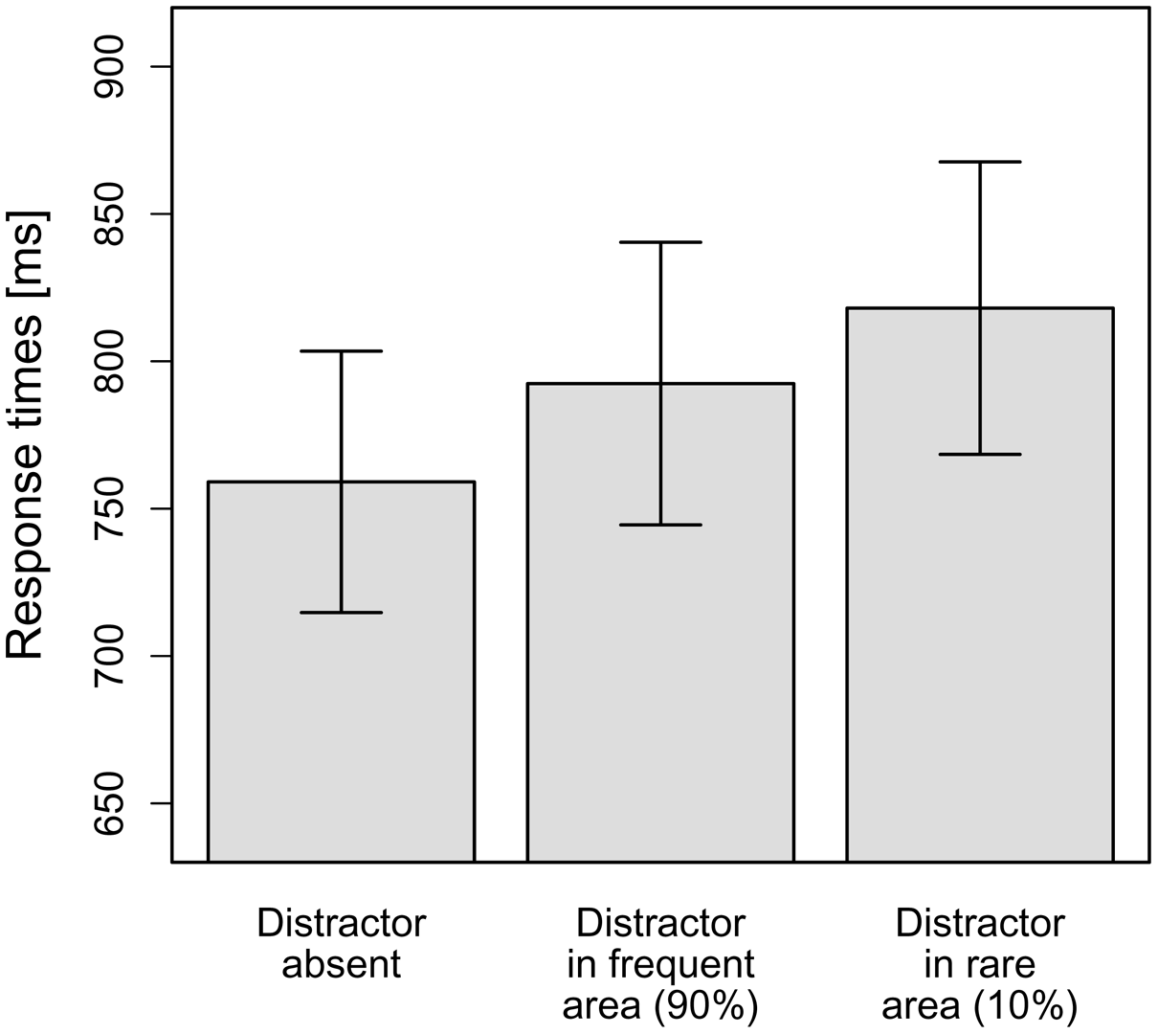
**Mean RTs dependent on the distractor condition in Experiment 3.** Error bars denote one standard error of the mean RT.

As can be seen in **Figure 4**, in line with our hypotheses, the interference caused by a distractor in the frequent hemifield (33 ms) was smaller than that caused by a distractor in the rare hemifield (59 ms). To statistically corroborate this observation, the mean RTs per observer and distractor condition (distractor-absent, distractor in frequent hemifield, distractor in rare hemifield) were subjected to a repeated-measure ANOVA. This revealed a significant main effect, *F*(1.31,24.87) = 15.16, MSE = 1,761.21, *p* < 0.001, $\eta_{p}^{2}$ = 0.44. Again, this effect was broken down by calculating planned (orthogonal, one-tailed) *t*-tests according to our hypotheses. The first comparison examined whether the distractors presented caused overall-interference. To this end, distractor-absent RTs were compared to the averaged RTs for distractors in the frequent and rare hemifields. As expected, RTs for distractor-absent trials were significantly faster compared to the mean of the two distractor-present conditions, *t*(19) = 5.91, *p* < 0.001, *d* = 1.32. The second comparison tested whether RTs were significantly faster if a distractor appeared in the frequent hemifield as compared to the rare hemifield – which was supported by the data, *t*(19) = –2.10, *p* = 0.025, *d* = 0.47. This means that even though distractor position repetitions were excluded by the design, Experiment 3 yielded comparable results to Experiment 1: interference by a salient but irrelevant distractor was reduced if it appeared at a frequent, as compared to a rare, distractor location. This supports the conclusion that the probability cueing effect observed in Experiment 1 is not only owing to facilitated suppression of distractors appearing at the same (repeated) position on consecutive trials, but is also driven by statistical learning, which takes place even when there are no distractor position repetitions^5^.

Finally, as in Experiment 1, we examined whether the reduced interference by distractors at frequent vs. rare locations is critically dependent on participants having “recognized” the distractor distribution. This analysis was based on participants’ responses to (post-experimental) queries 1 and 2. In response to the open query 1, only 1 out of the 20 participants reported that the distractor had appeared more frequently in one particular hemifield; in response to query 2 requiring a two-alternative forced-choice response, 13 participants (65%) indicated the frequent hemifield correctly; the others responded “don’t know”. Comparing performance between the two groups (13 correct responders vs. 7 “don’t know” responders in a group × distractor condition ANOVA revealed a significant main effect of distractor condition [*F*(1.30,23.38) = 13.03, *p* < 0.001; Greenhouse-Geisser-corrected values], but the theoretically critical interaction was not significant, *F*(1.30,23.38) = 0.18, *p* = 0.743 (Greenhouse-Geisser-corrected values). For participants unable to indicate the frequent distractor hemifield (“don’t know” responders), the singleton distractor produced an interference effect of 30 ms if it appeared at one of the frequent locations, and an effect of 60 ms if it appeared at one of the rare locations. This compares with effects of 40 ms and, respectively, 57 ms for participants who had correctly indicated the frequent distractor region. Thus, there was no evidence that the reduced interference for frequent as compared to rare positions was influenced by whether or not participants “recognized” the distractor distribution.

## GENERAL DISCUSSION

Taken together, the present results clearly demonstrate that there is a probability cueing effect for distractor locations: observers can take advantage of uneven spatial distributions of distracting objects to minimize interference by distractors at probable locations, as compared to distractors at less probable locations. The main goal of the present study was to examine the mechanism of this probability cueing modulation for distractor locations: do observers minimize interference by distractors in probable distractor positions because a distractor appearing at a probable position is more likely to appear at the position of a distractor on the previous trial (thus benefitting from intertrial facilitation) or is there an additional benefit of statistical learning of the spatial distractor distribution. To answer this question, we investigated both intertrial facilitation by distractor position repetitions and statistical learning of uneven spatial distractor distributions, independently of each other. Experiments 2 and 3 demonstrate that both of these factors yield a reduction of distractor interference – even in the absence of the respective other influencing factor. Experiment 2 showed that distractor position repetitions can lead to reduced distractor interference (as compared to distractor position switches) – despite the absence of an uneven spatial distractor distribution, that is, without any particular incentive to shield a recently encountered distractor position. Experiment 3, on the other hand, showed that uneven distractor distributions lead to reduced interference from distractors in probable areas, as compared to distractors in less probable areas – despite the absence of distractor position repetitions, that is, when intertrial facilitation effects are effectively prevented.

The observed individual benefits of intertrial facilitation (Experiment 2) and statistical learning (Experiment 3) were both smaller (in RT magnitude and effect size) than the combined effect observed in Experiment 1. In Experiment 2, repeating the distractor position (as compared to switching the distractor position) led to an interference reduction of about 26 ms (*d* = 0.64). In Experiment 3, a distractor in a frequent area caused 26 ms less interference than a distractor in a rare area (*d* = 0.47). In Experiment 1, the uneven spatial distractor distribution was confounded with distractor position repetitions, that is, both factors could contribute to the benefit for probable vs. less probable distractor regions. The observed benefit in this experiment was about 46 ms (*d* = 1.25), which corresponds roughly to the sum (52 ms) of the separate benefits caused by intertrial facilitation (Experiment 2) and statistical learning (Experiment 3) alone. Of course, these observations are insufficient for a strong claim of additivity but further argue that both statistical learning and intertrial priming contribute to the reduction of interference in Experiment 1.

The finding that both intertrial facilitation and statistical learning contribute to the probability cueing effect for distractor positions is in line with various other studies that examined probability cueing effects for target positions. For instance, Geng and Behrmann (2002, p. 1257) reported greater intertrial facilitation effects in a highly probable, as compared to a less probable, target region and thus concluded that there is “facilitation for high probability location targets over and above that of spatial repetition priming alone.” Druker and Anderson (2010) used continuous spatial target distributions across the display, thus creating a design that led to only very few spatial target repetitions. Nevertheless, they observed probability cueing effects and accordingly concluded that intertrial facilitation alone cannot account for probability cueing of target locations. On the other hand, there are reports claiming that probability cueing (for target locations) depends solely on intertrial facilitation (Walthew and Gilchrist, 2006) or that intertrial facilitation is a prerequisite for (additional) statistical learning effects to occur (Kabata and Matsumoto, 2012). This is not in line with the present results for distractor position probability cueing: statistical learning of distractor positions led to a probability cueing effect – even in the absence of distractor position repetitions (Experiment 3)^6^.

The present finding of reduced distractor interference for distractors in frequent (i.e., likely), as compared to rare (i.e., unlikely), distractor locations is also in line with findings demonstrating that endogenous cueing of a likely distractor location can be used to actively inhibit that location, thereby reducing interference by a distractor appearing there (Van der Stigchel and Theeuwes, 2006; Munneke et al., 2008). However, note that Jiang et al. (2013a) have recently compared the effects of endogenous cueing and statistical learning of target positions and concluded that the underlying attentional sources of those two effects are different. The present results demonstrate that not only endogenous cueing of likely distractor locations can be used to down-modulate interference by distractors appearing at these locations, but that probability cueing likewise has the potential to do so.

Investigating probability cueing of distractor locations (as opposed to probability cueing of target locations), and thus presenting singletons defined in two different feature dimensions, as in the present paradigm, may offer new insights into the potential mechanism underlying the probability cueing effect. On the one hand, probability cueing of locations might be a purely spatial mechanism, involving (coarse-grained) spatial suppression or, respectively, enhancement of visual coding. On the other hand, probability cueing might also involve a feature- or dimension-based component, that is, selectively influencing the processing of certain features or feature dimensions (at certain locations). The latter is a central component of Guided-Search-type models of visual attention (e.g., Wolfe et al., 1989; Wolfe, 1994; Müller et al., 1995; Found and Müller, 1996), which assume a processing architecture in which local feature contrast signals are first calculated in parallel (within separate dimensions). These signals can then be top–down modulated, or “weighted”, prior to their integration into a master salience map, which guides the deployment of attention. Hence, according to these models, the reduction of interference by salient, but irrelevant distractors might be owing to top–down up-weighting of the target-defining feature or feature dimension at the expense of the distractor-defining feature or feature dimension; or, likewise, to down-weighting (or “shielding”) of the distractor feature or dimension to the benefit of the target feature or dimension (e.g., Müller et al., 2009; Zehetleitner et al., 2012). To account for the present findings, such models would have to be extended by a spatial weighting component. For instance, it is conceivable that both feature-/dimension- and location-based weighing mechanisms may influence salience-based feature contrast signals prior to their integration into a master salience map (see Krummenacher et al., 2009, for a more detailed discussion of how these two mechanisms might interact).

## CONCLUSION

The present study investigated probability cueing of distractor locations and its underlying mechanisms. We demonstrate that observers can take advantage of an uneven spatial distribution of distractor locations to reduce interference by distractors at probable locations as compared to distractors at less probable locations – that is, probability cueing of distractor locations can serve as an effective attentional cue guiding the shielding of likely distractor locations, which is in line with the findings of Reder et al. (2003) and at variance with the findings of Kelley and Yantis (2009). We have identified both intertrial facilitation arising from repeating a distractor location and statistical learning independently of distractor position repetitions as (additively) contributing to the observed probability cueing modulation – in line with previous reports of probability cueing of target locations (e.g., Geng and Behrmann, 2005; Druker and Anderson, 2010).

## Conflict of Interest Statement

The authors declare that the research was conducted in the absence of any commercial or financial relationships that could be construed as a potential conflict of interest.

## Footnotes

1 One possibility, suggested to us by one of the reviewers, is that the distractor might have acted like an exogenous (automatic-reflexive) cue, given also that the distractor was slightly brighter than the target – with the “distractor” cue facilitating detection of targets on the “cued” vs. the “uncued”/opposite side. As can be seen from the description of the results above, both rare- and frequent-position distractors produced significant interference relative to the baseline – which was however, lower for frequent than for rare distractors (33 vs. 79 ms). Furthermore, as revealed by additional analyses, although there appeared to be a (statistically marginal) hemifield effect, this was due to distractors in the same hemifield as the target slowing RTs more than distractors in the opposite hemifield; this pattern was evident irrespective of whether distractors were rare [same vs. opposite hemifield: 809 vs. 774 ms; *t*(24) = 1.37, *p* = 0.183] or frequent [754 vs. 733 ms; *t*(24) = 1.72, *p* = 0.099]. This pattern would argue against the distractor – of whatever frequency – producing a (positive) cueing effect; rather, if anything, it would have produced a (negative) “localized attentional interference” (LAI) effect (Mounts, 2000, 2005; Mounts and Gavett, 2004), with targets in the vicinity of the distractor suffering more than targets further away. On LAI accounts, enhanced processing within the focus of attention is associated with a ring of suppression around the attended location (a “Mexican-hat”-type function; e.g., Hodgson et al., 1999; Müller et al., 2005; Caparos and Linnell, 2009), as a result of which items located within this ring are more difficult to detect. For a demonstration of this type of pattern in an additional singleton paradigm similar to that employed in the present study, see Koch et al. (2013). However, any such LAI effect would be largely additive to – that is, independent of – the distractor frequency effect revealed in Experiment 1. In any case, given that the spatial modulation of target detection by the distractor being in the same vs. a different hemifield is characteristic of the additional-singleton distractor producing attentional capture (Koch et al., 2013; see also Turatto et al., 2004; Hickey and Theeuwes, 2011), this would ultimately corroborate our interpretation of attentional capture being modulated by distractor position probability.

2 Given the thrust and design of Experiment 2, there was no post-experimental querying of participants’ explicit knowledge of distractor locations in Experiment 2.

3 To examine whether a similar pattern of cross-trial effects as found in Experiment 2 was also evident in Experiment 1, we examined for such effects in the data of Experiment 1 by comparing the following conditions: (1) distractor-absent on trial n; (2) distractor-present and distractor position repetition (relative to trial n-1); (3) distractor-present and distractor position change; (4) distractor-present on trial n, but absent on trial n-1. The RTs were 710, 733, 745, and 750 ms, respectively. Thus, Experiment 1 exhibited a similar trend as observed in Experiment 2, with RTs numerically faster for distractor position repetitions vs. changes. However, this effect was not significant in Experiment 1 [*t*(24) = –1.26, *p* = 0.219, two-tailed] – likely owing to the fact that there was a greater number of possible target and distractor positions compared to Experiment 2 (10 vs. 6), making direct position repetitions relatively rare. To further examine whether this difference would hold if the “direct” distractor position repetition vs. change criterion is relaxed to the distractor appearing in the same vs. the opposite hemifields on trials n-1 and n, the following conditions were compared: (1) distractor-absent on trial n; (2) distractor-present and in same hemifield as on trial n-1; (3) distractor-present and in opposite hemifield to trial n-1; (4) distractor-present on trial n, but absent on trial n-1. The RTs were 710, 736, 771, and 750 ms, respectively. Thus, indeed, interference is reduced on trials with a hemifield repetition as compared to trials with a hemifield change [736 vs. 771 ms; *t*(24) = 2.94, *p* = 0.007, two-tailed]. This would appear to suggest that cross-trial “priming of suppression” shows a degree of spreading, giving rise to “regional” – instead of just “direct-position” – suppression around the distractor position. Note, though, that this effect is, at least partially, confounded with frequent vs. rare distractor hemifield: since the distractor occurred more frequently in the “frequent” distractor hemifield, there were necessarily more hemifield repetitions for this hemifield. Accordingly, short-term adjustment vs. longer-term learning effects cannot be clearly separated.

4 The data of Experiment 2 were also analyzed as a function of the distractor condition on trial n-2. Overall, there was no significant difference in RTs between position repetition trials (from trial n-2 to trial n) and position switch trials (from trial n-2 to trial n), *t*(11) = –0.34, *p* = 0.74, *ns* (two-tailed). However, when only those trials were taken into account on which no distractor had been present on trial n-1, RTs were significantly faster for position repetitions from trial n-2 to trial n (*M* = 670 ms, SD = 137) than for position switches from trial n-2 to trial n (*M* = 710 ms, SD = 136), *t*(11) = –2.26, *p* = 0.045, *d* = 0.65 (two-tailed). Hence, in our paradigm, intertrial facilitation effects diminish after an intervening distractor-present trial, but may be carried over across intervening distractor-absent trials. Accordingly, in Experiment 3, we not only excluded distractor position repetitions from trial n-1 to trial n, but also from trials preceding trial n-1 if the intervening trials were all distractor-absent trials.

5 One interesting question, raised by one of the reviewers, concerns how the reduced interference from distractors at likely display locations does actually come about, in particular: does it reflect some kind of (general) spatial inhibition of likely distractor regions? To examine this, we compared, for Experiments 1 and 3, RTs to targets appearing in frequent vs. infrequent distractor regions on distractor-absent trials. For neither experiment was the effect significant, that is: targets were responded to equally rapidly (on distractor-absent trials) whether they appeared in a frequent or an infrequent distractor hemifield [Experiment 1: *t*(24) = –1.03, *p* = 0.312; Experiment 3: *t*(19) = –0.54, *p* = 0.597; numerically, 12 and, respectively, 5 ms faster RTs to targets in rare than in frequent distractor regions]. This outcome shows that the target, defined in a different dimension to the distractor, escapes the effect of frequent/rare distractor region, that is: this effect is not simply due to spatial inhibition of the likely distractor region on some general, attention-guiding spatial map, like the overall-saliency map. Instead, it might be the case that the spatial representation inhibited is dimension- (or even feature-) specific to the properties singling out the distractor amongst the other non-targets (see Zehetleitner et al., 2012, and see General Discussion). This, however, would need to be examined in further experiments.

6 Recall that in Experiment 3, as in Experiment 2, target and distractor positions were restricted to six positions on the middle circle of the stimulus display. Consequently, distractors on trial n also never appeared at a position directly adjacent to the position of a distractor on trial n-1, most likely ruling out the possibility of spreading positional inhibition contributing to the observed probability cueing effect.
